# Supplementary material for: A fault-intrusion-tolerant system and deadline-aware algorithm for scheduling scientific workflow in the cloud
Source: PeerJ Comput Sci. 2021 Nov 2;7:e747. doi: 10.7717/peerj-cs.747 (PMC8576554; doi:10.7717/peerj-cs.747)
Supplement: Supplemental Information 6 [file peerj-cs-07-747-s006.zip › Myproject1.1/docs/org/workflowsim/utils/package-tree.html]

org.workflowsim.utils Class Hierarchy


JavaScript is disabled on your browser.


- Overview
- Package
- Class
- Use
- Tree
- Deprecated
- Index
- Help

- Prev
- Next

- Frames
- No Frames

- All Classes

# Hierarchy For Package org.workflowsim.utils

Package Hierarchies:

- All Packages

## Class Hierarchy

- java.lang.Object
  - org.workflowsim.utils.ArgumentParser
  - org.workflowsim.utils.ClusteringParameters
  - org.workflowsim.utils.OverheadParameters
  - org.workflowsim.utils.Parameters
  - org.workflowsim.utils.ReplicaCatalog

## Enum Hierarchy

- java.lang.Object
  - java.lang.Enum<E> (implements java.lang.Comparable<T>, java.io.Serializable)
    - org.workflowsim.utils.Parameters.FTCMonitor
    - org.workflowsim.utils.Parameters.FTCMethod
    - org.workflowsim.utils.Parameters.FTCFailure
    - org.workflowsim.utils.ClusteringParameters.ClusteringMethod
    - org.workflowsim.utils.Parameters.SCHMethod
    - org.workflowsim.utils.ReplicaCatalog.FileSystem
    - org.workflowsim.utils.Parameters.PLNMethod

- Overview
- Package
- Class
- Use
- Tree
- Deprecated
- Index
- Help

- Prev
- Next

- Frames
- No Frames

- All Classes
